# Efficacy and Safety of Once-Weekly Lonapegsomatropin in Adults With Growth Hormone Deficiency: foresiGHt Trial Results

**DOI:** 10.1210/clinem/dgaf680

**Published:** 2025-12-20

**Authors:** Beverly M K Biller, Aleksandra Gilis-Januszewska, Mirjana Doknic, Antonio Miguel Pico, Maria Fleseriu, Gerald Raverot, Andrea M Isidori, Yutaka Takahashi, Jose M Garcia, Julie M Silverstein, Irina Bancos, Hidenori Fukuoka, Eric Huang, Jennifer Kang, Allison S Komirenko, Laurie Domrzalski, Aimee D Shu, Michael Beckert, Kevin C J Yuen

**Affiliations:** Neuroendocrine and Pituitary Tumor Clinical Center, Massachusetts General Hospital and Harvard Medical School, Boston, MA 02114, USA; Department of Endocrinology, Jagiellonian University, Medical College, 30-688 Kraków, Poland; University Clinical Center of Serbia, Medical Faculty University, 11000 Belgrade, Serbia; Clinical Medicine, Alicante Institute for Health and Biomedical Research (ISABIAL), 03202 Elche, Alicante, Spain; Department of Medicine and Neurological Surgery, Pituitary Center, Oregon Health and Science University, Portland, OR 97239, USA; Department of Endocrinology, Hospices Civils de Lyon, 69 500 Lyon Cedex 03, France; Department of Experimental Medicine, Sapienza University of Rome, 00185 Rome, Italy; Nara Medical University, Department of Diabetes and Endocrinology, Kashihara, Nara 634-8521, Japan; Department of Medicine, University of Washington/GRECC, Puget Sound VA, Seattle, WA 98108, USA; Division of Endocrinology, Metabolism & Lipid Research, Washington University School of Medicine, St Louis, MO 63110, USA; Division of Endocrinology, Diabetes, Metabolism and Nutrition, Mayo Clinic, Rochester, MN 55902, USA; Division of Diabetes and Endocrinology, Kobe University Hospital, Kobe 650-0017, Japan; Endocrine and Rare Disease Medical Sciences, Ascendis Pharma, Inc., Palo Alto, CA 94304, USA; Endocrine and Rare Disease Medical Sciences, Ascendis Pharma, Inc., Palo Alto, CA 94304, USA; Endocrine and Rare Disease Medical Sciences, Ascendis Pharma, Inc., Palo Alto, CA 94304, USA; Endocrine and Rare Disease Medical Sciences, Ascendis Pharma, Inc., Palo Alto, CA 94304, USA; Endocrine and Rare Disease Medical Sciences, Ascendis Pharma, Inc., Palo Alto, CA 94304, USA; Endocrine and Rare Disease Medical Sciences, Ascendis Pharma A/S, 2900 Hellerup, Denmark; Department of Neuroendocrinology and Neurosurgery, Barrow Neurological Institute, University of Arizona College of Medicine and Creighton University School of Medicine, Phoenix, AZ 85013, USA

**Keywords:** growth hormone, growth hormone deficiency, long-acting growth hormone, adult growth hormone deficiency

## Abstract

**Context:**

Adult growth hormone (GH) deficiency (GHD) is characterized by metabolic abnormalities caused by insufficient GH production. Lonapegsomatropin, a prodrug administered once weekly, was designed to provide sustained release of unmodified somatropin to reduce the burden of daily somatropin injections.

**Objective:**

This work aimed to evaluate the efficacy and safety of lonapegsomatropin vs placebo as treatment for adults with GHD.

**Methods:**

The foresiGHt trial was a multicenter, randomized, parallel-arm, placebo-controlled (double-blind) and active-controlled (open-label) trial (NCT04615273) conducted at 116 centers in North America, Europe, and Asia-Pacific. The trial randomly assigned and dosed 259 adults with GHD. Participants were randomly assigned 1:1:1 to receive once-weekly lonapegsomatropin, once-weekly placebo, or daily somatropin for 38 weeks. The primary efficacy end point was change from baseline in trunk percent fat at week 38. Secondary efficacy end points included change from baseline in trunk fat mass and total body lean mass.

**Results:**

At week 38, lonapegsomatropin significantly reduced trunk percent fat (−1.68% vs +0.37%; least squares [LS] mean difference −2.04%; *P* < .001), increased total body lean mass (+1.60 kg vs −0.11 kg; LS mean difference 1.70 kg; *P* < .0001), and reduced trunk fat mass (−0.48 kg vs +0.22 kg; LS mean difference −0.70 kg; *P* = .0053) vs placebo. The safety and tolerability profile of lonapegsomatropin was comparable to somatropin.

**Conclusion:**

The foresiGHt trial met its primary efficacy end point by demonstrating superiority of lonapegsomatropin vs placebo with similar safety and tolerability, supporting its potential as a once-weekly treatment option for adults with GHD.

Adults with growth hormone (GH) deficiency (GHD) experience an increase in body fat, particularly in the visceral compartment, reduced lean body mass, dyslipidemia, and insulin resistance, which predisposes this population to metabolic syndrome and an increased risk of cardiovascular morbidity and mortality (1-4). Additionally, this clinical syndrome is associated with impaired health-related quality of life (QoL), particularly cognitive dysfunction, depression, anxiety, sleep disturbance, fatigue, irritability, and reduced physical and mental drive (5, 6).

The primary treatment goal for treating GHD in adults is to restore GH levels to increase insulin-like growth factor-I (IGF-I) and improve the signs and symptoms in these patients (7-9). Previous studies have shown that daily somatropin replacement therapy reverses many features of GHD in adults, including decreasing body fat, increasing lean muscle mass, and improving QoL (10-14). Despite these clinical benefits, the published literature also indicates that adherence to daily somatropin replacement therapy in adults remains low (15, 16). Important factors identified as related to low adherence include perceived difficulty of injections, lack of choice of injection device, forgetting injections, and injection-related pain and discomfort (15, 17-19).

Treatment with somatropin has been the gold-standard treatment for patients with GHD for more than 25 years; however, daily injections are a known barrier to adherence. Long-acting GH products have recently been developed to address this challenge, with the potential to improve adherence and optimize clinical outcomes with less frequent injections while maintaining efficacy and safety comparable to daily somatropin (20).

Lonapegsomatropin (TransCon^®^ hGH [human GH]; SKYTROFA^®^), a prodrug of somatropin, is administered once weekly and designed to provide sustained release of active, unmodified somatropin (21). At physiologic pH and temperature, lonapegsomatropin releases somatropin via autocleavage of the TransCon linker in a predictable manner that follows first-order kinetics (21, 22). Somatropin released from lonapegsomatropin has the identical 191–amino acid sequence and size (22 kDa) as endogenous GH that binds to GH receptors found throughout the body (21). Lonapegsomatropin is currently approved in the United States, European Union, and other countries for the treatment of pediatric GHD (23-25).

Here, we present the results of the phase 3 foresiGHt trial, which evaluated the efficacy and safety of once-weekly lonapegsomatropin over 38 weeks in adults with GHD, highlighting its potential role as a GH replacement therapy for adults with GHD.

## Materials and Methods

### Trial Design and End Points

The foresiGHt trial (TCH-306) was a multicenter, randomized trial to evaluate the safety and efficacy of lonapegsomatropin in adults with GHD (ClinicalTrials.gov: NCT04615273; EudraCT: 2020-000929-42). The trial was double-blinded with respect to lonapegsomatropin and placebo, and open-label with respect to somatropin (Supplementary Fig. S1 (26)). The trial was conducted at 116 centers in North America (25 sites), Europe (60 sites), and Asia-Pacific (31 sites), with recruitment taking place from December 2020 to January 2023. Participants were randomly assigned in a 1:1:1 ratio to receive lonapegsomatropin, placebo, or somatropin (with stratification by dosing group, sex, and the presence of diabetes mellitus diagnosis at baseline). Following screening, the trial included a 38-week treatment period that consisted of a 12-week dose titration period followed by a 26-week dose maintenance period. Three dosing groups per treatment arm were defined based on participant age and concomitant use of oral estrogen.

The primary objective was to evaluate the efficacy of lonapegsomatropin compared to placebo at week 38. Secondary objectives included evaluation of the safety and tolerability, pharmacokinetics, and pharmacodynamics of lonapegsomatropin. An exploratory objective was to evaluate patient-reported outcomes (PROs). An additional exploratory objective was to evaluate the efficacy of once-weekly lonapegsomatropin compared with open-label daily somatropin.

The primary efficacy end point was the change from baseline in trunk percent fat at week 38. Secondary efficacy end points included change from baseline in trunk fat mass and total body lean mass; visceral adipose tissue was an exploratory end point. IGF-I SD score (SDS) was assessed as a pharmacodynamics end point. PROs were assessed by Treatment-Related Impact Measure–Adult Growth Hormone Deficiency (TRIM-AGHD) (27). Safety assessments included laboratory values, vital signs, electrocardiograms, fundoscopy, magnetic resonance imaging (at screening), adverse events (AEs), and treatment-emergent antibodies against lonapegsomatropin (prodrug), hGH (in the lonapegsomatropin and open-label somatropin arms), and released methoxypolyethylene glycol (mPEG; in the lonapegsomatropin arm).

### Participants and Trial Drug

Eligible participants were aged 23 to 80 years, inclusive, with biochemically confirmed GHD. For adult-onset GHD, a history of structural hypothalamic-pituitary disease, hypothalamic-pituitary surgery, cranial irradiation, additional pituitary hormone deficiencies, genetic etiology, or traumatic brain injury (with GHD confirmed by GH stimulation testing performed at least 12 months after the injury) was required. For childhood-onset GHD, persistent GHD must have been confirmed after achieving final height. Participants must have been naive to GH treatment or not been treated with GH within the prior 12 months. To ensure a relatively homogeneous trial population, IGF-I SDS at or below −1.0 (assessed by a central laboratory using the IDS-iSYS IGF-I assay (28)) was required at screening.

Participants requiring hormone replacement therapies (ie, glucocorticoids, thyroid hormone, estrogen, or testosterone) must have been treated with adequate and stable doses for 6 weeks or longer prior to and throughout screening. For participants not on glucocorticoid replacement therapy, adequate adrenal function (defined as morning serum cortisol greater than 15.0 µg/dL) and/or serum cortisol greater than 18.0 µg/dL on adrenocorticotropin stimulation test or insulin tolerance test was required. For men not on testosterone replacement therapy, morning total testosterone must have been within reference limits for age.

Key exclusion criteria were poorly controlled (hemoglobin A_1c_ [HbA_1c_] > 7.5%) or recently diagnosed (within 26 weeks) diabetes mellitus, active malignant disease or a history of malignancy (with certain exceptions), and evidence of growth of pituitary adenoma or other benign intracranial tumor within the last 12 months before screening. The complete eligibility criteria are listed in Supplementary Table S1 (26).

Lonapegsomatropin and placebo were provided as a lyophilized powder in single-use glass vials requiring reconstitution with sterile water for injection and administered as subcutaneous self-injections via syringe and needle. The placebo product contained the same excipients as the lonapegsomatropin drug product but not lonapegsomatropin itself. Somatropin was administered via a prefilled pen for daily subcutaneous self-injections.

Participants were initiated on a low dose of trial medication for 4 weeks, which was then increased at weeks 5 and 9 during the 12-week dose titration period (Supplementary Fig. S1 (26)). The dose titration period was then followed by an increase to the target maintenance dose at week 13, which was administered for 26 weeks to week 38 (dose maintenance period). The target maintenance doses were selected to ensure participants would receive adequate dosing for efficacy based on age and concomitant oral estrogen intake. Dose reduction or delay in dose escalation was permitted in cases of treatment-related AEs.

Adherence was assessed using participant diaries, which were completed on the day of each trial drug administration. Entries included the date and time of administration, dose, and injection site location. Trial staff reviewed the diaries at each visit to confirm adherence. Adherence was calculated as the number of doses administered divided by number of expected doses, multiplied by 100.

Investigators were blinded to IGF-I levels across the lonapegsomatropin, placebo, and open-label somatropin arms. In case of average weekly IGF-I SDS greater than 2.0, a dose reduction recommendation was provided by the unblinded team, separate from the sponsor. To maintain the blinding between lonapegsomatropin and placebo, sham dose reduction recommendations were conducted in the placebo arm in a pattern that followed the dose modifications of the lonapegsomatropin arm.

### Outcome Measures

Trunk percent fat, trunk fat mass, total body lean mass, and visceral adipose tissue (defined in Supplementary Fig. S2A (26)) were assessed using centrally read dual-energy x-ray absorptiometry (DXA) at baseline, at the end of the dose titration period, and at the end of the trial (week 38). Representative DXA images showing the trunk and visceral adipose tissue regions of interest are included in Supplementary Fig. S2B and S2C (26).

For IGF-I SDS, absolute values and changes from baseline at each visit were analyzed. At weeks 4, 8, 12, and 38, IGF-I was drawn 4 or 5 days after lonapegsomatropin dosing, corresponding to weekly average levels (29). At week 17, IGF-I was drawn 6 or 7 days after lonapegsomatropin dosing, corresponding to weekly trough level; at week 28, IGF-I was drawn 1 to 3 days after lonapegsomatropin dosing, corresponding to weekly peak level.

The safety analysis included incidence calculation of treatment-emergent AEs occurring in 5% or more of total participants in the safety population.

TRIM-AGHD is a disease-specific instrument that was used to assess the effect of GHD and its treatment on patients’ functioning and well-being (30). The TRIM-AGHD questionnaire covers physical health, cognitive ability, energy levels, and psychological health domains, with participant responses based on a 5-point Likert scale. For example, in response to “How often does your energy level interfere with what you can accomplish daily?”, possible responses range from 1 (“Never/almost never, Not at all bothered”) to 5 (“Almost always/always, Extremely bothered”). TRIM-AGHD is scored independently for each domain with score ranges of 0 to 100. Lower scores indicate a better health state, and a 10-point change in overall score is considered a clinically meaningful improvement (27). Additionally, an energy rating scale (unscored item) asks participants to rate their energy on a scale of 1 (“Extremely low energy”) to 5 (“Extremely high energy”). In this trial, the TRIM-AGHD questionnaire was completed by participants based on their experiences over the 2 weeks prior to completion. The TRIM-AGHD questionnaire was completed at baseline and at weeks 12, 28, and 38.

### Statistical Analysis

For the primary efficacy end point, the difference between lonapegsomatropin and placebo for change from baseline in trunk percent fat at week 38 was estimated using an ANCOVA model, with multiple imputation for missing data. The ANCOVA model included treatment arm, region (North America, Europe, or Asia-Pacific), baseline age group (< 30, 30-60, or > 60 years), sex, concomitant oral estrogen at screening in female participants (yes vs no), GHD onset (childhood vs adulthood), and baseline trunk percent fat as covariates. The open-label somatropin arm was included in the ANCOVA model of the primary analysis but was not powered for formal statistical comparison.

Subgroup analyses of the primary efficacy end point were performed to determine whether treatment effects were consistent across clinically meaningful subgroups. The difference in change from baseline at week 38 in trunk percent fat and their 95% CIs were displayed in a forest plot. Subgroups included the following: region, sex, GHD onset, and oral estrogen use prior to baseline.

For secondary efficacy end points, the analysis method used for the primary efficacy end point was applied with the corresponding baseline value as a covariate. A fixed-sequence testing procedure was applied to control the family-wise error rate at a level of .05. Under this testing procedure, the key secondary efficacy end points were tested only if superiority of the primary efficacy end point of trunk percent fat for lonapegsomatropin over placebo was met at a 2-sided .05 statistical significance level. If the *P* value for the primary end point was less than .05, then the 2 key secondary end points listed next were tested sequentially as follows:

- Test 1: change from baseline in total body lean mass at week 38; and
- Test 2: change from baseline in trunk fat mass at week 38 (tested only if the result of test 1 was statistically significant at *P* < .05).

Per protocol, average IGF-I SDS was to be maintained below 2.0 through dose reductions based on laboratory monitoring. However, due to a procedural oversight, some investigators did not receive timely notifications when IGF-I exceeded this threshold, resulting in higher-than-intended IGF-I levels in the lonapegsomatropin and open-label somatropin arms. To account for the higher-than-intended IGF-I SDS observed in the trial and to evaluate the relationship between IGF-I levels and treatment effects, a post hoc analysis was conducted to assess body composition changes in a subset of participants with IGF-I SDS less than or equal to 1.75 at week 38. This threshold aligns with the upper bound of the target IGF-I range used in a recent phase 3 adult GHD trial (31) and allows for more meaningful comparisons across the lonapegsomatropin and open-label somatropin arms at similar IGF-I exposures.

The normalized score for each of the domains in TRIM-AGHD (30, 32) was calculated; absolute values and change from baseline were summarized by treatment arm. The difference between treatment arms in change from baseline was performed using a similar ANCOVA model as that specified for the primary efficacy end point with baseline TRIM-AGHD score used as a covariate. A post hoc responder analysis at week 38 was performed using a Cochran-Mantel-Haenszel test controlling for dosing group. A participant was defined as a responder if the change from baseline in TRIM-AGHD total score decreased by 10 points or more, corresponding to the minimal important difference defined for TRIM-AGHD (27). The total score was calculated as the mean of nonmissing normalized score among the Physical Health, Cognitive Ability, Energy Levels, and Psychological Health domains.

### Statement of Ethics

The trial was approved by appropriate institutional review boards and independent ethics committees of each participating site. The trial was conducted in accordance with the principles of the Declaration of Helsinki, Good Clinical Practice as described by the International Conference on Harmonization Guidelines, and applicable local regulations. All participants provided written informed consent prior to enrollment.

## Results

### Baseline Characteristics

**Figure 1.**
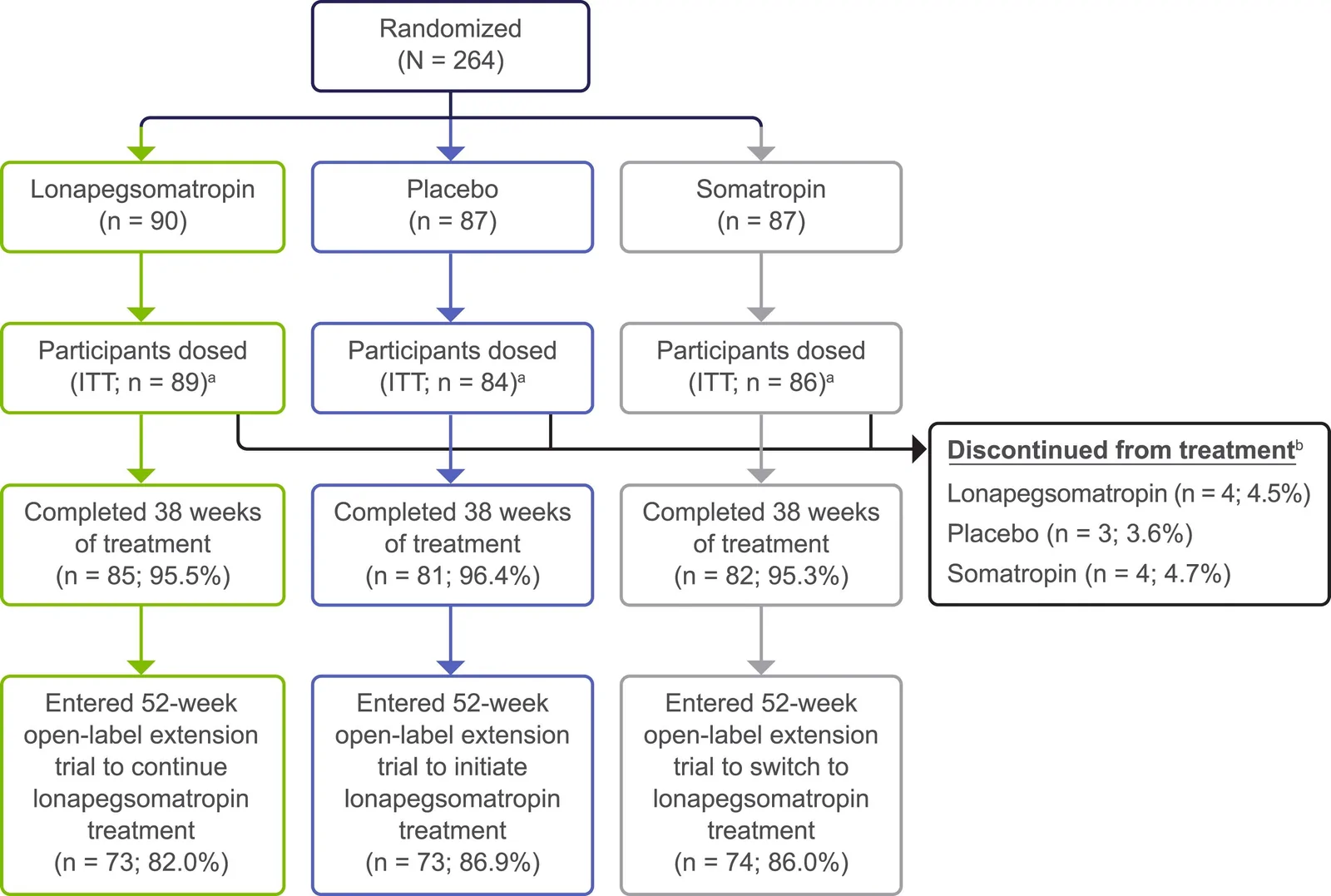
Participant disposition. ^a^Five randomly assigned participants were not dosed and were not included in the ITT population. ^b^Reasons for discontinuation of treatment in each arm included the following: lonapegsomatropin (patient withdrew [n = 3], adverse event [epilepsy; n = 1]); placebo (lost to follow-up [n = 1], patient withdrew [n = 1], other [patient changed country of residence; n = 1]); somatropin (patient withdrew [n = 2], physician decision [n = 1], adverse event [transitional cell carcinoma; n = 1]). ITT, intent-to-treat population.

A total of 259 participants were randomly assigned and dosed in the trial: there were 89 who received lonapegsomatropin, 84 received placebo, and 86 received somatropin (Fig. 1). A total of 248 (95.8%) participants completed the foresiGHt trial, and of those, 220 (88.7%) continued onto the 52-week open-label extension trial (TCH-306EXT).

Demographics and baseline characteristics were generally well balanced across the treatment arms (Table 1). The trial population had a mean (SD) age of 42.8 (14.2) years and comprised slightly more male (54.1%) than female participants. The majority of participants (51.7%) were in the dosing group for participants aged 30 to 60 years without oral estrogen intake (Supplementary Table S2 (26)). Adult-onset GHD was reported for 56.0% of participants, with the remaining 44.0% having childhood-onset GHD; the mean duration since GHD diagnosis was 15.3 years (range, 0.10-52.84 years).

**Table 1.** Demographics and baseline characteristics (intent-to-treat population)

	Lonapegsomatropin (n = 89)	Placebo (n = 84)	Somatropin (n = 86)	Total (N = 259)
**Age, mean (SD), y**	43.0 (13.4)	44.1 (14.7)	41.3 (14.3)	42.8 (14.2)
**GHD onset, n (%)**				
Adult	50 (56.2)	46 (54.8)	49 (57.0)	145 (56.0)
Childhood	39 (43.8)	38 (45.2)	37 (43.0)	114 (44.0)
**Pituitary deficiencies, n (%)**				
GHD and additional hormone deficiencies	84 (94.4)	79 (94.0)	83 (96.5)	246 (95.0)
Adrenal deficiency	70 (78.7)	66 (78.6)	68 (79.1)	204 (78.8)
Thyroid deficiency	78 (87.6)	75 (89.3)	78 (90.7)	231 (89.2)
Gonadal deficiency	78 (87.6)	70 (83.3)	78 (90.7)	226 (87.3)
Vasopressin deficiency	20 (22.5)	31 (36.9)	26 (30.2)	77 (29.7)
GHD only	5 (5.6)	5 (6.0)	3 (3.5)	13 (5.0)
Panhypopituitarism	77 (86.5)	73 (86.9)	78 (90.7)	228 (88.0)
**Female, n (%)**	42 (47.2)	39 (46.4)	38 (44.2)	119 (45.9)
On oral estrogen*^a^*	21 (23.6)	16 (19.0)	18 (20.9)	55 (21.2)
**Etiology of GHD,*^b^* n (%)**				
Hypothalamic-pituitary surgery	36 (40.4)	31 (36.9)	31 (36.0)	98 (37.8)
Pituitary tumor	27 (30.3)	28 (33.3)	29 (33.7)	84 (32.4)
Structural hypothalamic-pituitary defect	15 (16.9)	21 (25.0)	14 (16.3)	50 (19.3)
Idiopathic	14 (15.7)	8 (9.5)	13 (15.1)	35 (13.5)
Genetic*^c^*	6 (6.7)	5 (6.0)	7 (8.1)	18 (6.9)
Cranial irradiation	3 (3.4)	5 (6.0)	8 (9.3)	16 (6.2)
Traumatic brain injury	2 (2.2)	2 (2.4)	2 (2.3)	6 (2.3)
Other*^d^*	6 (6.7)	6 (7.1)	7 (8.1)	19 (7.3)
**BMI ≥ 30, n (%)**	21 (23.6)	35 (41.7)	34 (39.5)	90 (34.7)

Abbreviations: BMI, body mass index; GHD, growth hormone deficiency.

^*a*^Percentages based on all participants.

^*b*^Categories not mutually exclusive.

^*c*^Genetic mutations were on *PROP1*, *PROKR2*, and *GH-N*.

^*d*^Other includes lymphocytic hypophysitis, Langerhans cell histiocytosis, pituitary apoplexy, pituitary gland necrosis, and Sheehan syndrome.

A variety of etiologies for GHD were recorded, with the most common being hypothalamic-pituitary surgery (37.8%) and pituitary tumor (32.4%). Nearly all (94.2%) participants had additional pituitary hormone deficiencies, including 89.2% with thyroid deficiency, 87.3% with gonadal deficiency, 78.8% with adrenal deficiency, and 29.7% with vasopressin deficiency. Most participants (88.0%) had panhypopituitarism, defined as deficiencies in two or more pituitary axes in addition to GHD, with prevalence by treatment arm of 86.5% in the lonapegsomatropin group, 86.9% in the placebo group, and 90.7% in the somatropin group. Among female participants, nearly half (55/119 women; 21.2% of the overall trial population) were on oral estrogen therapy. Vitamin D deficiency (18.5%), obesity (14.7%), hypertension (13.5%), osteopenia (8.1%), depression (8.1%), osteoporosis (6.9%), gastroesophageal reflux disease (6.6%), and lipid abnormalities (32.8%; including dyslipidemia [15.8%], hyperlipidemia [10.4%], and hypercholesterolemia [6.6%]) were among the most common comorbidities reported across all participants.

At baseline, the mean (SD) IGF-I SDS was −2.68 (1.07) for the total population. Across the lonapegsomatropin, placebo, and open-label somatropin arms, the mean (SD) body mass index (BMI) was 27.0 (5.0), 28.5 (6.5), and 28.6 (7.2), respectively. Approximately 35% of participants had a BMI in the obese category (≥ 30), including 23.6% of patients treated with lonapegsomatropin, 41.7% with placebo, and 39.5% with somatropin. Approximately 4% had diabetes mellitus.

### Dosing and Adherence

Treatment adherence was high, with 91.0%, 94.0%, and 89.4% of participants in the lonapegsomatropin, placebo, and open-label somatropin arms, respectively, having adherence rates between 90% and 100%. Over 38 weeks, a similar amount of GH was administered in the lonapegsomatropin and open-label somatropin arms, but with fewer injections in the weekly lonapegsomatropin arm than in the open-label somatropin arm (mean 36.7 injections vs 250.2 injections, respectively).

In the dose maintenance period (weeks 13-38), 29.5% of participants underwent dose adjustments. The most common reason for dose adjustment during the maintenance period was IGF-I SDS monitoring (22.9% of trial participants), during which doses were reduced in response to average IGF-I SDS above 2.0.

### Body Composition

**Figure 2.**
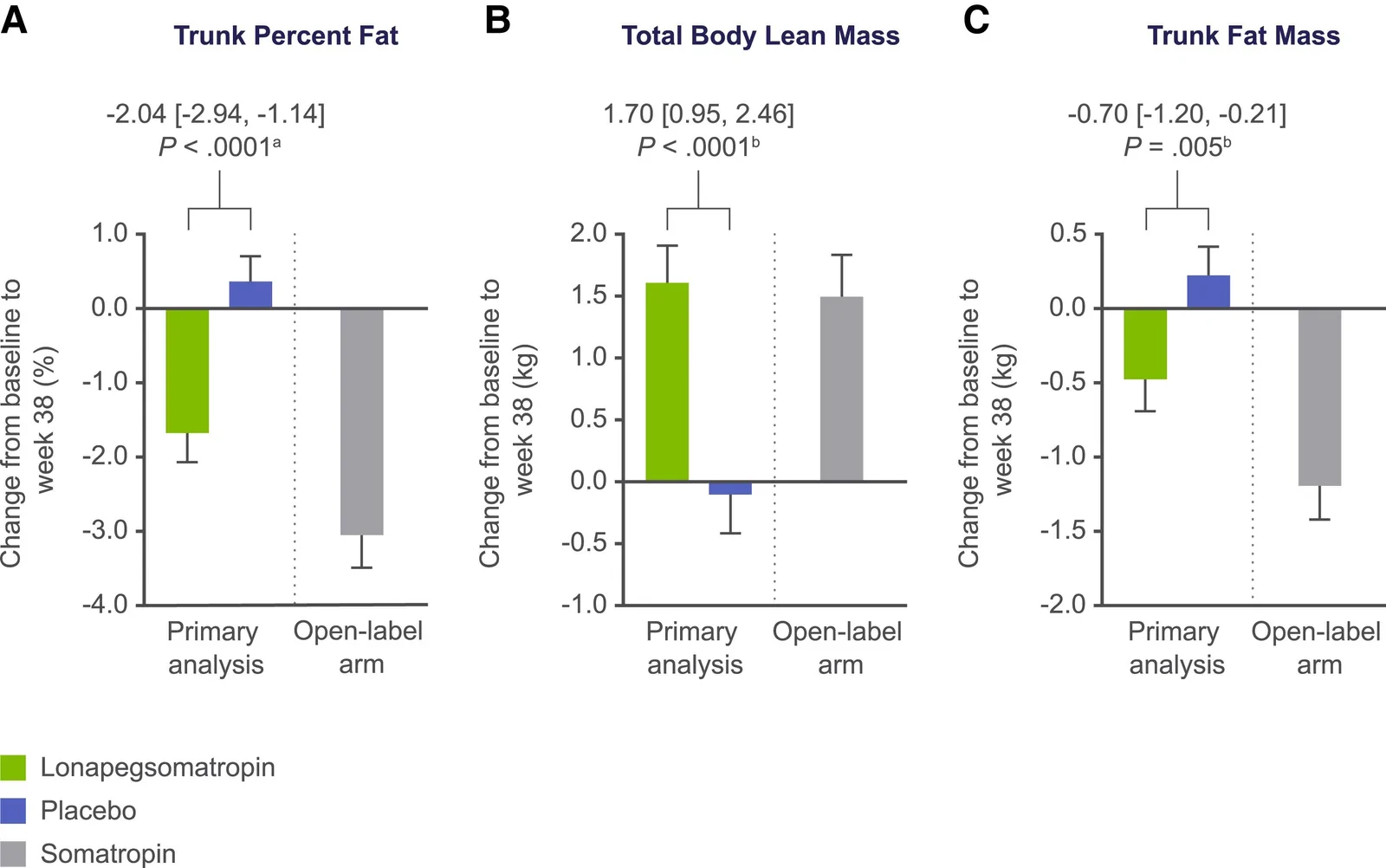
Body composition change from baseline at week 38 (intent-to-treat population). Change from baseline at week 38 for A, trunk percent fat; B, total body lean mass; and C, trunk fat mass. Data are presented as the LS mean. Error bars represent SE. The difference in change from baseline at week 38 was estimated using an ANCOVA model including treatment arm, region, baseline age group, sex, concomitant oral estrogen at screening in female participants, GHD onset, and baseline value of the end point as covariates. The LS mean difference in change from baseline at week 38 for lonapegsomatropin vs placebo and 95% CIs are shown. No formal statistical comparisons were conducted between the lonapegsomatropin and somatropin groups. ^a^Primary efficacy end point. ^b^Secondary efficacy end point. ANCOVA, analysis of covariance; GHD, growth hormone deficiency; LS, least squares; SE, standard error.

Lonapegsomatropin treatment reduced trunk percent fat from baseline at 38 weeks compared to placebo (−1.68% vs placebo +0.37%, respectively; LS mean difference −2.04%; 95% CI, −2.94 to −1.14; *P* < .0001; Fig. 2A). Greater increases in total body lean mass (lonapegsomatropin +1.60 kg vs placebo −0.11 kg; LS mean difference 1.70 kg, 95% CI, 0.95-2.46; *P* < .0001) (Fig. 2B) and reductions in trunk fat mass (lonapegsomatropin −0.48 kg vs placebo +0.22 kg; LS mean difference −0.70 kg, 95% CI, −1.20 to −0.21; *P* = .005) (Fig. 2C) were observed with lonapegsomatropin relative to placebo at week 38.

**Figure 3.**
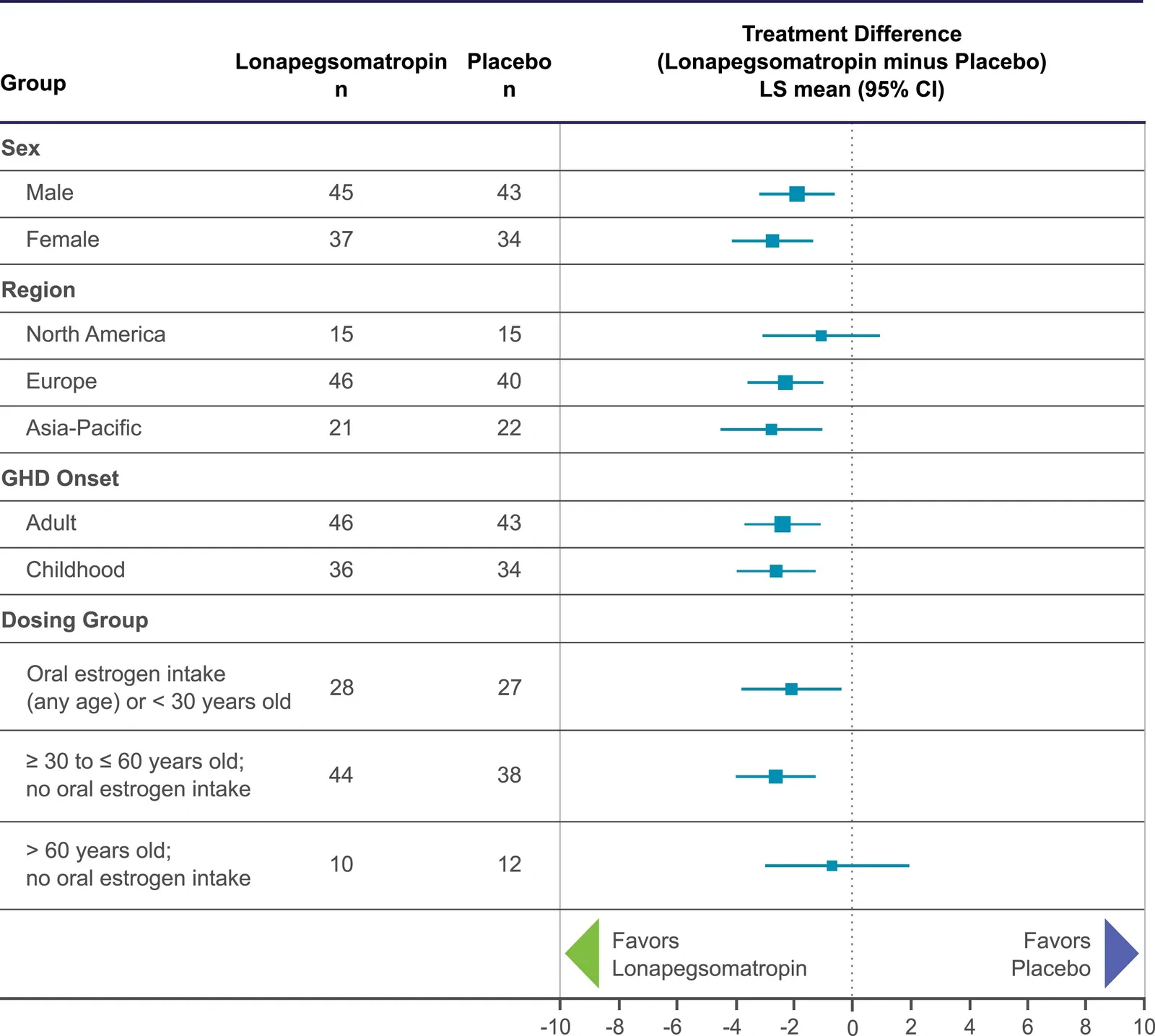
Change from baseline in trunk percent fat at week 38 by subgroups (intent-to-treat population). Forest plot of least squares (LS) mean ± CIs of treatment difference (from ANCOVA model) for change from baseline in trunk percent fat at 38 for all evaluable patients with dual-energy x-ray absorptiometry measurement at week 38. ANCOVA, analysis of covariance; GHD, growth hormone deficiency.

Subgroup analyses demonstrated that the LS mean treatment difference favoring lonapegsomatropin vs placebo for change from baseline in trunk percent fat at week 38 was maintained across subgroups, including sex, region, GHD onset, and dosing group, as shown in Fig. 3.

In exploratory efficacy analyses, also depicted in Fig. 2, the LS mean change from baseline to week 38 in the open-label somatropin arm showed similar directional trends as for lonapegsomatropin, with reductions in trunk percent fat (−3.05%; see Fig. 2A), increases in total body lean mass (+1.49 kg; see Fig. 2B), and reductions in trunk fat mass (−1.20 kg; see Fig. 2C).

For the exploratory end point of visceral adipose tissue, lonapegsomatropin demonstrated a decrease at week 38 compared with placebo (LS mean difference −0.10 kg; *P* = .0034). The open-label somatropin arm also showed a decrease in visceral adipose tissue (LS mean −0.13 kg) at week 38.

### Pharmacodynamics

**Figure 4.**
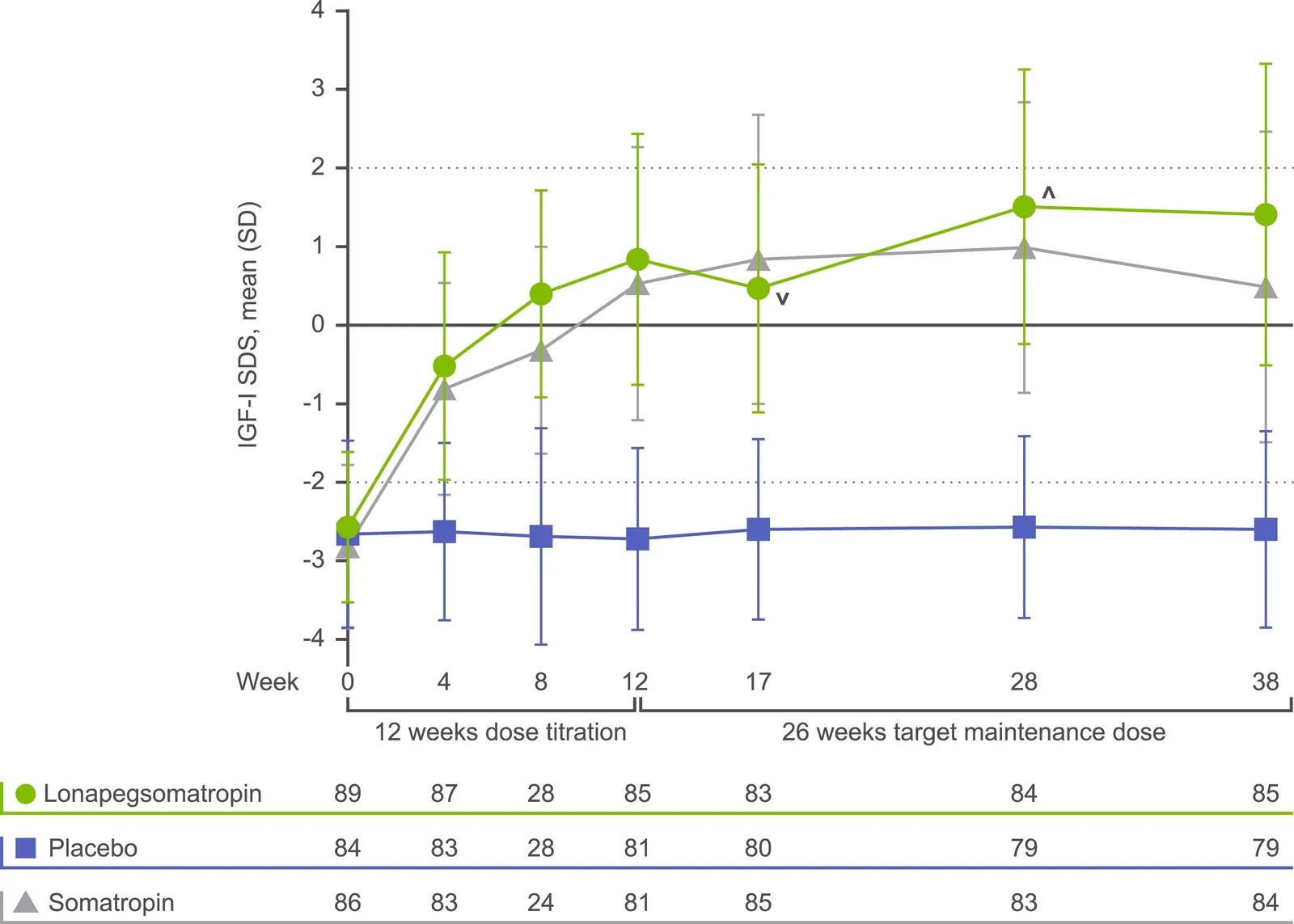
IGF-I SDS by visit (pharmacokinetics/pharmacodynamics analysis set). At weeks 4, 8, 12, and 38, IGF-I was drawn 4 or 5 days after lonapegsomatropin dosing, corresponding to weekly average levels. _∨_Denotes IGF-I drawn 6 or 7 days after lonapegsomatropin dosing, corresponding to weekly trough levels. ^∧^Denotes IGF-I drawn 1 to 3 days after lonapegsomatropin dosing, corresponding to weekly peak levels. IGF-I SDS values are reported as sampled. IGF-I, insulin-like growth factor I; SD, standard deviation; SDS, standard deviation score.

IGF-I SDS increased from baseline in lonapegsomatropin-treated participants, with mean (SD) of 1.41 (1.92) at week 38 (Fig. 4). As expected, IGF-I SDS in the placebo arm remained relatively unchanged throughout the trial, with a mean (SD) of −2.60 (1.25) at week 38. The LS mean (SE) change in IGF-I SDS from baseline to week 38 was 4.01 (0.20) for lonapegsomatropin. At week 38, 54.1% (46 of 85 participants) had IGF-I SDS within the reference range (−2 to +2 SDS); 5.9% (5 of 85 participants) and 40.0% (34 of 85 participants) had IGF-I SDS below and above the reference range, respectively.

In somatropin-treated participants, mean (SD) IGF-I SDS was 0.49 (1.98) at week 38 (see Fig. 4). In the open-label somatropin arm, IGF-I was inadvertently measured more than 24 hours after the last dose in 25 of 84 participants (29.8%). Among the 59 (70.2%) participants in the open-label somatropin arm with IGF-I collected within 24 hours after last dose at week 38, the mean (SD) of IGF-I SDS at week 38 was 1.11 (1.97). Overall, in the open-label somatropin arm, the LS mean (SE) change in IGF-I SDS from baseline to week 38 was 3.31 (0.22). At week 38, 21.4% (18 of 84 participants) of the somatropin participants had IGF-I SDS above 2.0.

### Post Hoc Analysis of Body Composition in Lonapegsomatropin- and Somatropin-Treated Participants With Comparable Therapeutic Insulin-Like Growth Factor-I Levels

**Figure 5.**
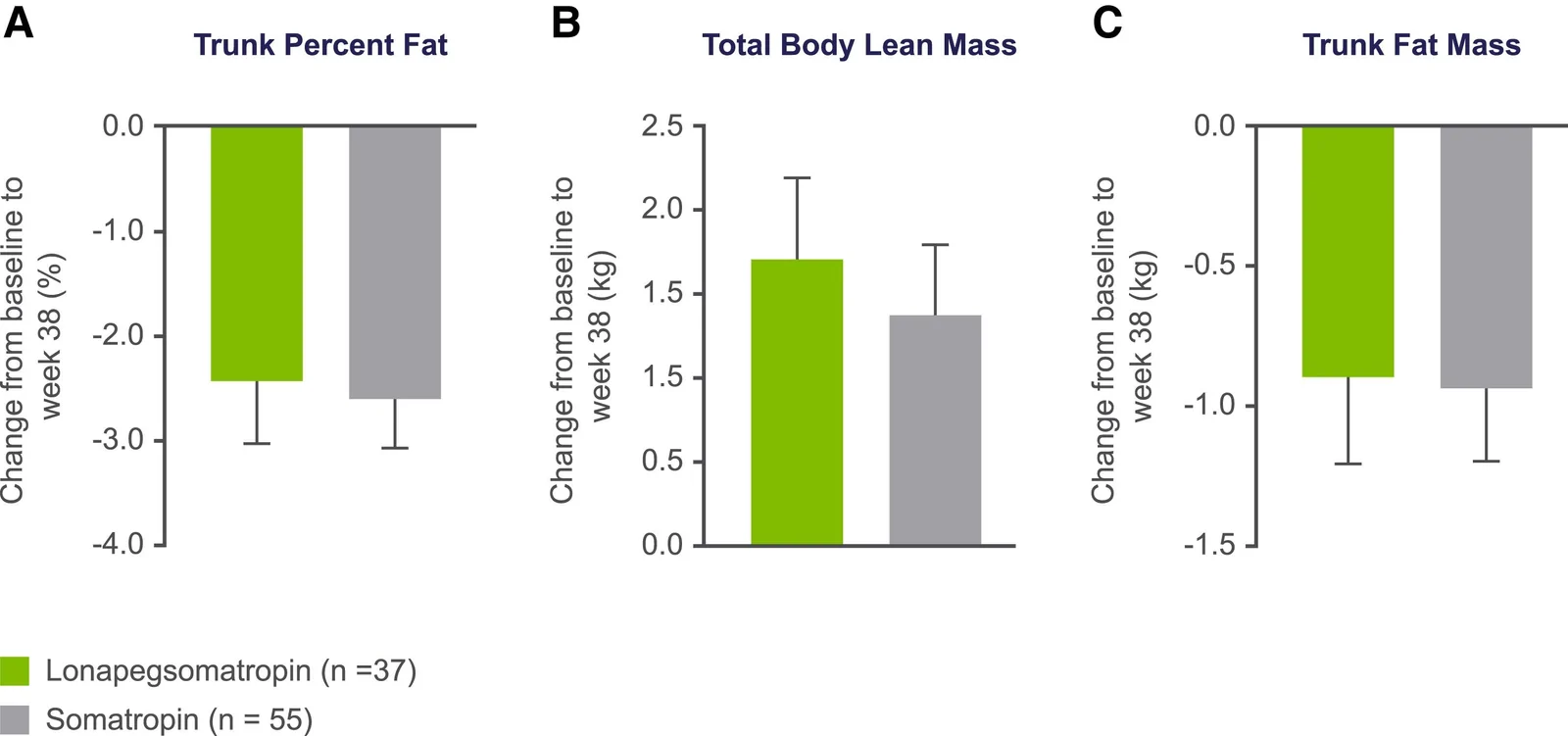
Body composition change from baseline at week 38 in participants with comparable IGF-I SDS. Post hoc analyses for change from baseline in A, trunk percent fat; B, trunk fat mass; and C, total body lean mass at week 38 in participants with observed IGF-I SDS less than or equal to 1.75 at week 38. Data are presented as least squares mean (SE). The difference in change from baseline at week 38 was estimated using an analysis of covariance model including treatment arm, region, baseline age group, sex, concomitant oral estrogen at screening in female patients, GHD onset, and baseline value of the end point as covariates. GHD, growth hormone deficiency; IGF-I, insulin-like growth factor I; SDS, standard deviation score; SE, standard error.

For participants with IGF-I SDS less than or equal to 1.75 at week 38, changes in trunk percent fat were similar between lonapegsomatropin (−2.42%, n = 37) and somatropin (−2.59%, n = 55) (Fig. 5A). Increases in total lean mass were similar between the 2 treatment arms, with an LS mean change of +1.70 kg in the lonapegsomatropin arm compared to +1.37 kg in the open-label somatropin arm (Fig. 5B). Similarly, reductions in trunk fat mass were nearly identical, with an LS mean change of −0.90 kg for lonapegsomatropin and −0.94 kg for somatropin (Fig. 5C). Similar trends were also observed in more stringent IGF-I SDS subsets (≤1.5 and ≤ 1.25; data not shown) at week 38.

### Patient-Reported Outcomes

Based on TRIM-AGHD, participants reported a reduction in burden of their GHD and treatment across physical health, cognitive ability, energy levels, and psychological health for the lonapegsomatropin, placebo, and open-label somatropin arms. At week 38, change from baseline LS mean (SE) values were as follows: for physical health, −8.69 (2.05), −5.71 (2.17), and −8.91 (2.18); for cognitive ability, −6.34 (1.84), −5.71 (2.18), and −3.47 (1.77); for energy levels, −3.70 (3.10), −2.69 (2.73), and −2.38 (2.85); and for psychological health, −3.47 (1.22), −1.38 (1.42), and −1.72 (1.49), corresponding to the lonapegsomatropin, placebo, and open-label somatropin arms, respectively.

Additionally, an increase in energy from baseline to week 38 was reported by participants for the lonapegsomatropin, placebo, and open-label somatropin arms, with an LS mean (SE) for the normalized score on the energy scale of +8.07 (2.52), +6.31 (2.40), and +5.57 (2.34), respectively.

The percentage of participants with an improvement of 10 points (minimal important difference (27)) in total score was 39.2%, 29.3%, and 28.8% in the lonapegsomatropin, placebo, and open-label somatropin arms, respectively.

### Safety and Tolerability

A similar overall incidence of treatment-emergent AEs (∼70%) was observed across the lonapegsomatropin, placebo, and open-label somatropin arms (Table 2). There were no deaths and no participants discontinued study drug due to an AE assessed as related by the investigator. The most common AEs (> 5% of the total population) were COVID-19, arthralgia, nasopharyngitis, headache, upper respiratory tract infection, and injection site reactions. AEs considered related to study drug (as assessed by the investigator) were reported in 24.7% of lonapegsomatropin-treated participants, 13.1% of placebo-treated participants, and 22.1% of open-label somatropin-treated participants (see Table 2).

**Table 2.** Adverse events (safety population)

	Lonapegsomatropin (n = 89)	Placebo (n = 84)	Somatropin (n = 86)
Treatment-emergent AEs, n (%)	64 (71.9)	55 (65.5)	63 (73.3)
Severity*^a^*			
Mild, n (%)	37 (41.6)	31 (36.9)	36 (41.9)
Moderate, n (%)	24 (27.0)	23 (27.4)	25 (29.1)
Severe, n (%)	3 (3.4)	1 (1.2)	2 (2.3)
Related AEs, n (%)	22 (24.7)	11 (13.1)	19 (22.1)
Serious AEs, n (%)	4 (4.5)	1 (1.2)	6 (7.0)
Serious and related AEs, n (%)	1 (1.1)	0	1 (1.2)
Deaths, n (%)	0	0	0
AE that led to study drug discontinuation, n (%)	1 (1.1)	0	1 (1.2)
AE that led to any action on study drug, n (%)	8 (9.0)	1 (1.2)	11 (12.8)
AEs occurring in ≥ 5% of all participants, n (%)			
Arthralgia	8 (9.0)	8 (9.5)	7 (8.1)
COVID-19	7 (7.9)	11 (13.1)	6 (7.0)
Nasopharyngitis	5 (5.6)	11 (13.1)	6 (7.0)
Headache	7 (7.9)	9 (10.7)	5 (5.8)
Injection site reaction*^b^*	4 (4.5)	4 (4.8)	5 (5.8)
Upper respiratory tract infection	2 (2.2)	8 (9.5)	4 (4.7)

Abbreviation: AE, adverse event.

^*a*^In the severity categories, participants are displayed for the highest severity only.

^*b*^ *Injection site* reaction is a combined term that includes preferred terms of injection site erythema, bruising, pain, hematoma, hemorrhage, pruritus, and atrophy. All injection site reactions were mild or moderate in severity.

Serious adverse events (SAEs) occurred in 11 participants overall, including 4 (4.5%) participants in the lonapegsomatropin arm, 1 (1.2%) participant in the placebo arm, and 6 (7.0%) participants in the open-label somatropin arm. Two participants (0.8%) overall experienced SAEs assessed by the investigator as related to the study drug: One lonapegsomatropin-treated participant (1.1%) was hospitalized for moderate hyponatremia (treatment was temporarily interrupted), and one somatropin-treated participant (1.2%) was hospitalized for moderate facial and peripheral edema. Two participants discontinued treatment due to unrelated SAEs: One participant in the lonapegsomatropin arm experienced a single epileptic seizure in the setting of a preexisting ventriculoperitoneal shunt, and one participant in the open-label somatropin arm was diagnosed with transitional cell carcinoma during the trial. The remaining serious events were noncardiac chest pain (n = 1; 1.1%) and coronavirus pneumonia (n = 1; 1.1%) in the lonapegsomatropin arm; acute kidney injury (n = 1; 1.2%) in the placebo arm; and seizure (n = 1; 1.2%), drug eruption due to astaxanthin (n = 1; 1.2%), anemia (n = 1; 1.2%), and hypertension (n = 1; 1.2%) in the open-label somatropin arm.

Severe AEs occurred in 6 participants: 3 (3.4%) in the lonapegsomatropin arm, 1 (1.2%) in the placebo arm, and 2 (2.3%) in the open-label somatropin arm. Two events, the single epileptic seizure in the lonapegsomatropin arm and seizure in the open-label somatropin arm, were also classified as serious (as described earlier). The remaining severe AEs were increased γ-glutamyltransferase and gout in the lonapegsomatropin arm, traumatic intracranial hemorrhage in the placebo arm, and arthralgia in the open-label somatropin arm. Of the severe events, only arthralgia (reported in the open-label somatropin arm) was considered related to the study drug by the investigator.

Overall, no clinically meaningful differences or patterns in glucose metabolism parameters (insulin, fasting glucose, HbA_1c_) were found between lonapegsomatropin-, placebo-, and somatropin-treated participants. Of the 11 participants with diabetes mellitus at baseline, 4 had adjustments to their diabetes pharmacotherapy—2 due to adverse events, including diabetic metabolic decompensation or increased HbA_1c_, and 2 based on their medical history of diabetes; 2 participants were not on any diabetes medications prior to or throughout the trial; and the remaining 5 had no changes to their diabetes medication regimen during the trial. One participant in the open-label somatropin arm was diagnosed with diabetes mellitus during the trial; no new-onset diabetes mellitus was reported in the lonapegsomatropin or placebo arms. As with participants from the placebo and open-label somatropin arms, participants treated with lonapegsomatropin showed stable mean levels of lipid, glycemic, hematology, chemistry, hormonal, renal, and hepatic parameters over time, for which the mean values remained within normal reference ranges. Mean values for vital signs and electrocardiogram assessments remained within normal limits throughout the study across the trial population.

The incidence of treatment-emergent antibodies (combined antilonapegsomatropin, anti-hGH, or anti-mPEG antibodies) was low (3.4%) in lonapegsomatropin-treated participants. All detected antibodies were low titer (≤ 80) and transient (detected only once or twice, < 4 months apart). No anti-hGH antibodies were detected in participants treated with somatropin. No neutralizing antibodies were detected in participants treated with lonapegsomatropin or somatropin.

## Discussion

This phase 3 foresiGHt trial met its primary efficacy end point, demonstrating superiority of once-weekly lonapegsomatropin over placebo in reducing trunk percent fat at week 38 of treatment. Compared to placebo, lonapegsomatropin also reduced trunk fat mass and visceral adipose tissue while increasing total body lean mass at week 38, reflecting its efficacy in improving body composition in adults with GHD. These changes support a more balanced body composition profile, with GH-driven effects that may reflect benefits in overall endocrine and metabolic health. Additionally, lonapegsomatropin treatment increased IGF-I levels, with a mean IGF-I SDS value of 1.41 at week 38, within the reference range of −2.0 to +2.0. Overall, comparable safety and tolerability was observed in the trial for lonapegsomatropin as compared with somatropin. These outcomes are consistent with the known physiologic and metabolic effects observed with daily GH replacement therapy and support the clinical utility of lonapegsomatropin as a treatment option for adults with GHD.

Although the trial was not powered for formal comparisons between lonapegsomatropin and somatropin, changes in body composition in the open-label lonapegsomatropin arm reflected the same directional trends observed in the somatropin arm, reinforcing the metabolic efficacy of once-weekly lonapegsomatropin therapy.

To achieve adequate and equivalent weekly GH exposure across treatment arms in a clinical trial setting, dosing tables were used in this trial and gave rise to a broad range of IGF-I values, reflecting individual variability in GH responsiveness. As GH is lipolytic and IGF-I is adipogenic (33), a post hoc analysis was conducted to better understand body composition changes for similar IGF-I levels. In the IGF-I SDS less than or equal to 1.75 subset analysis, reductions in trunk percent fat and trunk fat, and increases in lean mass, were similar between lonapegsomatropin and somatropin, suggesting that when IGF-I exposure is comparable, the metabolic effects of these therapies are well aligned. These findings were expected given that lonapegsomatropin releases unmodified somatropin that binds to the GH receptors. The results also highlight that for tissues in which GH and IGF-I act synergistically (such as epiphyses—relevant for pediatric GHD—and muscle), comparable effects can be seen across the dosing spectrum; whereas for tissues in which GH (lipolytic) and IGF-I (lipogenic) have opposing effects (such as in fat), comparable effects may be limited to a dosing or IGF-I range below a certain threshold. This is clinically relevant, as clinicians typically titrate the GH dose in adults with GHD to maintain IGF-I within −2.0 to +2.0 SDS (8).

A recent randomized, placebo-controlled trial in adults with GHD treated with US Food and Drug Administration–approved somapacitan showed the efficacy and safety of a long-acting GH product, with a reduction of −1.06% in trunk percent fat compared to an increase of +0.47% in the placebo arm, resulting in a treatment difference of −1.53% (31). In the present trial, lonapegsomatropin demonstrated a treatment difference of −2.04% compared with placebo for change from baseline in trunk percent fat at week 38. Additionally, in a post hoc analysis, lonapegsomatropin demonstrated a comparable treatment effect to open-label somatropin on target tissues. For participants with IGF-I SDS less than or equal to 1.75 at week 38, reductions in trunk percent fat (−2.42% lonapegsomatropin vs −2.59% somatropin) and trunk fat mass (−0.90 kg lonapegsomatropin vs −0.94 kg somatropin), with simultaneous increase in total body lean mass (+1.70 kg lonapegsomatropin vs +1.37 kg somatropin) were observed. Lonapegsomatropin provides a long-acting GH treatment option with favorable effects on body composition.

Several single-arm studies have demonstrated improvement in QoL in adults with GHD receiving GH replacement therapy (34-36). In this trial, numerical improvements were observed in the TRIM-AGHD questionnaire, particularly for physical health and cognitive ability, for lonapegsomatropin. A greater proportion of participants receiving lonapegsomatropin achieved a clinically meaningful improvement—defined as a 10-point increase in total TRIM-AGHD score—compared with those receiving placebo, although the difference was not statistically significant. These findings may suggest meaningful patient-perceived benefits with lonapegsomatropin in adults with GHD.

Safety was comparable between lonapegsomatropin and somatropin, and no new safety signals for lonapegsomatropin emerged. AE incidence was similar across treatment arms, and common events included COVID-19, arthralgia, nasopharyngitis, and headache. Notably, injection site reactions were mild to moderate with comparable incidence across lonapegsomatropin, placebo, and somatropin arms (4.5%, 4.8%, and 5.8%, respectively), regardless of administration method, with somatropin delivered via pen device and lonapegsomatropin administered using a vial and syringe in this trial. Eleven participants (4.2%) had type 2 diabetes mellitus at baseline. Importantly, across all trial participants, glucose metabolism parameters, including fasting glucose and HbA_1c_, remained stable throughout the trial, and no new-onset diabetes mellitus was observed in the lonapegsomatropin arm. Two SAEs (one in the lonapegsomatropin arm and one in the somatropin arm) were deemed related to the study drug, both of which resolved without long-term sequelae. Antibody incidence was low, and no neutralizing antibodies to lonapegsomatropin were detected.

The observed normalization of IGF-I and body composition improvements in the foresiGHt trial are clinically meaningful, as they reflect the physiologic benefits of GH replacement and the potential to correct the specific hormone deficiency underlying the clinical manifestations of GHD. In addition to its metabolic effects, once-weekly lonapegsomatropin offers greater convenience compared to daily GH therapy and may improve adherence by virtue of reductions in injection frequency (20)—an important consideration for adults with GHD who undergo long-term therapy and often are on multiple other medications for comorbid conditions.

This trial has several strengths. It was a global, large, multicenter trial conducted across 21 countries, supporting broad relevance of the findings. The trial was rigorously designed, featuring a randomized, double-blind design for the lonapegsomatropin and placebo arms. Participant retention was high throughout the 38-week treatment period, supporting the reliability of longitudinal assessments, with 85% of participants electing to roll over into the extension trial. Importantly, there were no deaths reported in the safety population. Deaths, particularly related to unrecognized or undertreated adrenal insufficiency, have occurred in other phase 3 adult GHD trials (31, 37), highlighting the importance of comprehensive monitoring and management of this complex patient population.

The trial also had several limitations. The 38-week duration of treatment may limit the ability to fully characterize the long-term efficacy and safety of lonapegsomatropin, particularly for outcomes such as body composition and QoL, which may require longer follow-up to capture the full therapeutic effect. While these data are not reported in the present manuscript, this study was followed by a 52-week open-label extension trial, which allows for up to 90 weeks of total treatment in participants who continue. The open-label extension may provide additional insights into longer-term outcomes when these data become available. Additionally, while the open-label somatropin arm provided useful context, comparisons between lonapegsomatropin and somatropin were not the primary objective of the trial; hence no formal hypothesis testing was planned for the comparisons between the lonapegsomatropin and open-label somatropin arms. A future analysis of the open-label extension trial will provide valuable data on long-term safety, adherence, and metabolic outcomes. While dosing tables were used in this clinical trial to achieve equivalent dosing across arms, IGF-I–based titration is used in clinical practice with a goal of maintaining IGF-I levels within the normal range.

In conclusion, once-weekly lonapegsomatropin significantly improved key measures of body composition (fat and lean tissue) in adults with GHD and was generally well tolerated. Lonapegsomatropin may represent an efficacious, safe, and convenient treatment option for adults with GHD.

## Data Availability

The datasets generated and/or analyzed during the current study are not publicly available but are available for noncommercial, academic purposes from the sponsor, absent legal reasons to the contrary, on reasonable request.

## Clinical Trial Information

ClinicalTrials.gov registration number NCT04615273 (registered October 29, 2020); EudraCT registration number 2020-000929-42 (registered June 9, 2021).

## Prior Publication

These data were previously presented as an oral presentation at ENDO 2024, Boston, Massachusetts, June 1 to 4, 2024, and at the Joint Congress of ESPE and ESE 2025, Copenhagen, Denmark, May 10 to 13, 2025.

## Abbreviations

AEs: adverse events
ANCOVA: analysis of covariance
BMI: body mass index
DXA: dual-energy x-ray absorptiometry
GH: growth hormone
GHD: growth hormone deficiency
HbA_1c_: hemoglobin A_1c_
hGH: human growth hormone
IGF-I: insulin-like growth factor-I
LS: least squares
mPEG: methoxypolyethylene glycol
PROs: patient-reported outcomes
QoL: quality of life
SAEs: serious adverse events
SD: standard deviation
SDS: standard deviation score
SE: standard error
TRIM-AGHD: Treatment-Related Impact Measure–Adult Growth Hormone Deficiency

## References

[1] Attanasio AF, Mo D, Erfurth EM, et al Prevalence of metabolic syndrome in adult hypopituitary growth hormone (GH)-deficient patients before and after GH replacement. J Clin Endocrinol Metab. 2010;95(1):74‐81. 10.1210/jc.2009-1326 19897679

[2] Gazzaruso C, Gola M, Karamouzis I, Giubbini R, Giustina A. Cardiovascular risk in adult patients with growth hormone (GH) deficiency and following substitution with GH—an update. J Clin Endocrinol Metab. 2014;99(1):18‐29. 10.1210/jc.2013-2394 24217903

[3] Aversa LS, Cuboni D, Grottoli S, Ghigo E, Gasco V. A 2024 update on growth hormone deficiency syndrome in adults: from guidelines to real life. J Clin Med. 2024;13(20):6079. 10.3390/jcm13206079 PMC11508958 39458028

[4] Fukuoka H, Endo T, Tsuboi S, Fujio S. Prevalence and risk of complications in untreated patients with adult growth hormone deficiency. Pituitary. 2025;28(2):32. 10.1007/s11102-025-01500-9 PMC11836217 39966200

[5] Loftus J, Camacho-Hubner C, Hey-Hadavi J, Goodrich K. Targeted literature review of the humanistic and economic burden of adult growth hormone deficiency. Curr Med Res Opin. 2019;35(6):963‐973. 10.1080/03007995.2018.1546682 30411985

[6] Fleseriu M, Christ-Crain M, Langlois F, Gadelha M, Melmed S. Hypopituitarism. Lancet. 2024;403(10444):2632‐2648. 10.1016/S0140-6736(24)00342-8 38735295

[7] Brod M, Pohlman B, Højbjerre L, Adalsteinsson JE, Rasmussen MH. Impact of adult growth hormone deficiency on daily functioning and well-being. BMC Res Notes. 2014;7(1):813. 10.1186/1756-0500-7-813 PMC4255661 25406443

[8] Yuen KCJ, Biller BMK, Radovick S, et al American Association of Clinical Endocrinologists and American College of Endocrinology guidelines for management of growth hormone deficiency in adults and patients transitioning from pediatric to adult care. Endocr Pract. 2019;25(11):1191‐1232. 10.4158/GL-2019-0405 31760824

[9] Molitch ME, Clemmons DR, Malozowski S, Merriam GR, Vance ML. Evaluation and treatment of adult growth hormone deficiency: an Endocrine Society clinical practice guideline. J Clin Endocrinol Metab. 2011;96(6):1587‐1609. 10.1210/jc.2011-0179 21602453

[10] Møller N, Jørgensen JO. Effects of growth hormone on glucose, lipid, and protein metabolism in human subjects. Endocr Rev. 2009;30(2):152‐177. 10.1210/er.2008-0027 19240267

[11] Newman CB, Carmichael JD, Kleinberg DL. Effects of low dose versus high dose human growth hormone on body composition and lipids in adults with GH deficiency: a meta-analysis of placebo-controlled randomized trials. Pituitary. 2015;18(3):297‐305. 10.1007/s11102-014-0571-z 24810900

[12] Snyder PJ, Biller BM, Zagar A, et al Effect of growth hormone replacement on BMD in adult-onset growth hormone deficiency. J Bone Miner Res. 2007;22(5):762‐770. 10.1359/jbmr.070205 17280527

[13] Burman P, Broman JE, Hetta J, et al Quality of life in adults with growth hormone (GH) deficiency: response to treatment with recombinant human GH in a placebo-controlled 21-month trial. J Clin Endocrinol Metab. 1995;80(12):3585‐3590. 10.1210/jcem.80.12.8530603 8530603

[14] van Bunderen CC, Deijen JB, Drent ML. Effect of low-normal and high-normal IGF-1 levels on memory and wellbeing during growth hormone replacement therapy: a randomized clinical trial in adult growth hormone deficiency. Health Qual Life Outcomes. 2018;16(1):135. 10.1186/s12955-018-0963-2 PMC6035403 29980224

[15] Rosenfeld RG, Bakker B. Compliance and persistence in pediatric and adult patients receiving growth hormone therapy. Endocr Pract. 2008;14(2):143‐154. 10.4158/EP.14.2.143 18308651

[16] Hoffman AR, Raveendran S, Manjelievskaia J, et al Prevalence, treatment patterns, and characteristics of US adults with confirmed or at risk for growth hormone deficiency. Adv Ther. 2025;42(6):2853‐2873. 10.1007/s12325-025-03188-6 PMC12085356 40261564

[17] Auer MK, Stieg MR, Hoffmann J, Stalla GK. Is insulin-like growth factor-I a good marker for treatment adherence in growth hormone deficiency in adulthood? Clin Endocrinol. 2016;84(6):862‐869. 10.1111/cen.13030 26824335

[18] Mancini A, Vergani E, Bruno C, et al The adult growth hormone multicentric retrospective observational study: a 24-month Italian experience of adherence monitoring via Easypod™ of recombinant growth hormone treatment in adult GH deficiency. Front Endocrinol. 2023;14:1298775. 10.3389/fendo.2023.1298775 PMC10666163 38027149

[19] Kirsch S, Butler G, Jensen LF, Okkels A, Yssing C, Håkan-Bloch J. Utilities associated with the treatment of growth hormone deficiency (GHD): a time trade-off (TTO) study in the UK and Canada. Patient Relat Outcome Meas. 2025;16:9‐21. 10.2147/PROM.S479705 PMC11731022 39811679

[20] Christiansen JS, Backeljauw PF, Bidlingmaier M, et al Growth Hormone Research Society perspective on the development of long-acting growth hormone preparations. Eur J Endocrinol. 2016;174(6):C1‐C8. 10.1530/EJE-16-0111 PMC5081743 27009113

[21] Thornton PS, Maniatis AK, Aghajanova E, et al Weekly lonapegsomatropin in treatment-naive children with growth hormone deficiency: the phase 3 heiGHt trial. J Clin Endocrinol Metab. 2021;106(11):3184‐3195. 10.1210/clinem/dgab529 PMC8530727 34272849

[22] Chatelain P, Malievskiy O, Radziuk K, et al A randomized phase 2 study of long-acting TransCon GH vs daily GH in childhood GH deficiency. J Clin Endocrinol Metab. 2017;102(5):1673‐1682. 10.1210/jc.2016-3776 28201598

[23] SKYTROFA (lonapegsomatropin-tcgd) [package insert]. Ascendis Pharma; July 2025. https://www.ascendispharma.us/products/pi/skytrofa/skytrofa_pi.pdf

[24] Ascendis Pharma Endocrinology Division A/S . SKYTROFA EPAR – Product Information. 2022. Accessed August 1, 2025. https://www.ema.europa.eu/en/documents/product-information/skytrofa-previously-lonapegsomatropin-ascendis-pharma-epar-product-information_en.pdf

[25] Therapeutic Goods Administration . Skytrofa (lonapegsomatropin-tcgd) – Product Information. Therapeutic Goods Administration. Accessed June 23, 2025. https://www.ebs.tga.gov.au/ebs/picmi/picmirepository.nsf/pdf?OpenAgent=&id=CP-2025-PI-01619-1&d=20250623172310101

[26] Biller BMK, Gilis-Januszewska A, Doknic M, et al Supplementary materials for “Efficacy and Safety of Once-Weekly Lonapegsomatropin in Adults With Growth Hormone Deficiency: foresiGHt Trial Results”. Figshare. 2025. 10.6084/m9.figshare.30983071 PMC13183427 41420532

[27] Brod M, Beck JF, Højbjerre L, et al Assessing the impact of growth hormone deficiency (GHD) in adults: interpreting change of the treatment-related impact measure-adult growth hormone deficiency (TRIM-AGHD). Pharmacoecon Open. 2019;3(1):71‐80. 10.1007/s41669-018-0082-3 PMC6393279 29797004

[28] Bidlingmaier M, Friedrich N, Emeny RT, et al Reference intervals for insulin-like growth factor-1 (igf-i) from birth to senescence: results from a multicenter study using a new automated chemiluminescence IGF-I immunoassay conforming to recent international recommendations. J Clin Endocrinol Metab. 2014;99(5):1712‐1721. 10.1210/jc.2013-3059 24606072

[29] Lin Z, Shu AD, Bach M, Miller BS, Rogol AD. Average IGF-1 prediction for once-weekly lonapegsomatropin in children with growth hormone deficiency. J Endocr Soc. 2022;6(1):bvab168. 10.1210/jendso/bvab168 PMC8668201 34913019

[30] Brod M, Højbjerre L, Adalsteinsson JE, Rasmussen MH. Assessing the impact of growth hormone deficiency and treatment in adults: development of a new disease-specific measure. J Clin Endocrinol Metab. 2014;99(4):1204‐1212. 10.1210/jc.2013-3438 24438372

[31] Johannsson G, Gordon MB, Højby Rasmussen M, et al Once-weekly somapacitan is effective and well tolerated in adults with GH deficiency: a randomized phase 3 trial. J Clin Endocrinol Metab. 2020;105(4):e1358‐e1376. 10.1210/clinem/dgaa049 PMC7076631 32022863

[32] Brod M, Hojbjerre L, Alolga SL, Beck JF, Wilkinson L, Rasmussen MH. Understanding treatment burden for children treated for growth hormone deficiency. Patient. 2017;10(5):653‐666. 10.1007/s40271-017-0237-9 PMC5605605 28386679

[33] Ranke MB, Savage MO, Chatelain PG, Preece MA, Rosenfeld RG, Wilton P. Long-term treatment of growth hormone insensitivity syndrome with IGF-I. Results of the European multicentre study. The working group on growth hormone insensitivity syndromes. Horm Res. 1999;51(3):128‐134. 10.1159/000023345 10461018

[34] Webb SM . Measurements of quality of life in patients with growth hormone deficiency. J Endocrinol Invest. 2008:31(9 Suppl):52‐55. 19020387

[35] Elbornsson M, Horvath A, Götherström G, Bengtsson B-Å, Johannsson G, Svensson J. Seven years of growth hormone (GH) replacement improves quality of life in hypopituitary patients with adult-onset GH deficiency. Eur J Endocrinol. 2017;176(2):99‐109. 10.1530/EJE-16-0875 27803031

[36] Mo D, Blum WF, Rosilio M, Webb SM, Qi R, Strasburger CJ. Ten-year change in quality of life in adults on growth hormone replacement for growth hormone deficiency: an analysis of the hypopituitary control and complications study. J Clin Endocrinol Metab. 2014;99(12):4581‐4588. 10.1210/jc.2014-2892 25233155

[37] Hoffman AR, Biller BM, Cook D, et al Efficacy of a long-acting growth hormone (GH) preparation in patients with adult GH deficiency. J Clin Endocrinol Metab. 2005;90(12):6431‐6440. 10.1210/jc.2005-0928 16159930

